# Minocycline, a microglial inhibitor, blocks spinal CCL2-induced heat hyperalgesia and augmentation of glutamatergic transmission in substantia gelatinosa neurons

**DOI:** 10.1186/1742-2094-11-7

**Published:** 2014-01-10

**Authors:** Chung-Yu Huang, Ying-Ling Chen, Allen H Li, Juu-Chin Lu, Hung-Li Wang

**Affiliations:** 1 Department of Physiology and Pharmacology, School of Medicine, Chang Gung University, 259 Wen-Hwa 1 road, Kwei-San, Tao-Yuan 333, Taiwan; 2 Chang Gung University of Science and Technology, 261 Wen-Hwa 1 road, Kwei-San, Tao-Yuan 333, Taiwan; 3 Department of Anesthesiology, Chang Gung Memorial Hospital, 5 Fu-Shing St, Kwei-San, Tao-Yuan 333, Taiwan; 4 Healthy Aging Research Center, Chang Gung University, Kwei-San, Tao-Yuan, Taiwan; 5 Neuroscience Research Center, Chang Gung Memorial Hospital, Kwei-San, Tao-Yuan, Taiwan

**Keywords:** CC chemokine ligand 2, Heat hyperalgesia, Substantia gelatinosa neurons, Glutamatergic transmission, Microglia, Tumor necrosis factor-α

## Abstract

**Background:**

Several lines of evidence suggest that CCL2 could initiate the hyperalgesia of neuropathic pain by causing central sensitization of spinal dorsal horn neurons and facilitating nociceptive transmission in the spinal dorsal horn. The cellular and molecular mechanisms by which CCL2 enhances spinal pain transmission and causes hyperalgesia remain unknown. The substantia gelatinosa (lamina II) of the spinal dorsal horn plays a critical role in nociceptive transmission. An activated spinal microglia, which is believed to release pro-inflammatory cytokines including TNF-α, plays an important role in the development of neuropathic pain, and CCL2 is a key mediator for spinal microglia activation. In the present study, we tested the hypothesis that spinal CCL2 causes the central sensitization of substantia gelatinosa neurons and enhances spinal nociceptive transmission by activating the spinal microglia and augmenting glutamatergic transmission in lamina II neurons.

**Methods:**

CCL2 was intrathecally administered to 2-month-old male rats. An intrathecal injection of CCL2 induced heat hyperalgesia, which was assessed using the hot plate test. Whole-cell voltage-clamp recordings substantia gelatinosa neurons in spinal cord slices were performed to record glutamatergic excitatory postsynaptic currents (EPSCs) and GABAergic inhibitory postsynaptic currents (IPSCs).

**Results:**

The hot plate test showed that 1 day after the intrathecal injection of CCL2 (1 μg), the latency of hind-paw withdrawal caused by a heat stimulus was significantly reduced in rats. One day after the intrathecal administration of CCL2, the amplitude of the evoked glutamatergic EPSCs and the frequency of spontaneous glutamatergic miniature EPSCs (mEPSCs) were significantly increased in outer lamina II neurons. Intrathecal co-injection of minocycline, a specific inhibitor of microglial activation, and CCL2 blocked the CCL2-induced reduction in the latency of hind-paw withdrawal and thermal hyperalgesia. Following intrathecal co-administration of CCL2 and minocycline, CCL2 failed to increase the frequency of glutamatergic mEPSCs and failed to promote glutamine release in lamina II neurons. Intrathecal co-injection of WP9QY, a selective TNF-α antagonist, and CCL2 completely inhibited CCL2-induced heat hyperalgesia and inhibited the increase in the frequency of glutamatergic mEPSCs in substantia gelatinosa neurons.

**Conclusion:**

In summary, our results suggest that an intrathecal injection of CCL2 causes thermal hyperalgesia by augmenting the excitatory glutamatergic transmission in substantia gelatinosa neurons through a presynaptic mechanism and facilitating nociceptive transmission in the spinal dorsal horn. Further studies show that intrathecal co-administration of minocycline, a specific inhibitor of microglial activation, or WP9QY, a selective TNF-α antagonist, completely inhibited CCL2 potentiation of glutamatergic transmission in substantia gelatinosa neurons and CCL2-induced heat hyperalgesia. The results of the present study suggest that peripheral nerve injury-induced upregulation of the spinal CCL2 level causes the central sensitization of substantia gelatinosa neurons by activating spinal microglia and that TNF-α mediates CCL2-induced thermal hyperalgesia and augmentation of glutamatergic transmission in lamina II neurons.

## Background

Several lines of evidence indicate that CC chemokine ligand 2 (CCL2)/monocyte chemoattractant protein-1 released during peripheral nerve injury is involved in mediating the hyperalgesia of neuropathic pain in the spinal dorsal horn [1-7]. CCL2 is constitutively present in dorsal root ganglion (DRG) neurons with small or medium diameters and their processes in the dorsal horn of the spinal cord (DHSC), in which it is co-localized with transient receptor potential vanilloid receptor 1 (TRPV1) and the pain-related peptide, substance P (SP), or calcitonin gene-related peptide (CGRP) [2]. The presence of CCL2 in primary nociceptive afferent fibers and in secretory vesicles in the DHSC suggests that CCL2 is transported and subsequently released from the central terminals of nociceptive DRG neurons [2,5,8]. In accord with this hypothesis, high potassium or capsaicin levels evoke a calcium-dependent secretion of CCL2 from DHSC explants of rats [2,5]. The expression level of CCL2 or its receptor CCR2 in the spinal dorsal horn is upregulated in animal models of neuropathic pain [1,3,4,9,10]. Furthermore, a higher level of CCL2 released from the DHSC has been observed in a rat model of neuropathic pain [5]. When administered intrathecally into naive rats, CCL2 induces thermal hyperalgesia [2,11]. In addition, an intrathecal administration of anti-CCL2 antibodies inhibits neuropathic pain behavior [12]. These findings suggest that CCL2 could initiate the hyperalgesia of neuropathic pain by causing central sensitization of spinal dorsal horn neurons and enhancing nociceptive transmission in the spinal dorsal horn. Further study is required to investigate the cellular and molecular mechanisms by which CCL2 facilitates pain transmission in the spinal dorsal horn.

Finely myelinated A*δ* and unmyelinated C glutamatergic afferent fibers that convey predominantly nociceptive information terminate within the substantia gelatinosa (lamina II) of spinal dorsal horn [13-17]. Pain information carried by small primary afferent fibers is processed and integrated by lamina II nociceptive neurons. Therefore, the substantia gelatinosa functions as the first relay station in the nociceptive pathway and plays a critical role in nociceptive transmission [16,17]. The excitability and activity of lamina II neurons is mainly regulated by glutamate released from primary nociceptive afferents [14,16,17]. Under pathological conditions, augmented glutamatergic transmission and resulting enhanced excitability of substantia gelatinosa neurons is expected to cause the hyperalgesia [18,19]. Immunohistochemical staining of CCL2 is most intense within the substantia gelatinosa, and CCL2 also co-localizes with substance P and CGRP-immunoreactive axon terminals in the outer portion of lamina II [2]. Following peripheral nerve injury, central axon terminals of DRG neurons and activated astrocytes in the spinal dorsal horn release CCL2 [3,5]. Considering the physiological importance of lamina II neurons in processing pain sensation, it is very likely that during neuropathic pain, upregulated CCL2 in superficial dorsal horn induces the hyperalgesia by augmenting excitatory glutamatergic transmission of substantia gelatinosa neurons and causing the central sensitization of lamina II neurons.

Finely myelinated A*δ* and unmyelinated C glutamatergic afferent fibers that convey predominantly nociceptive information terminate within the substantia gelatinosa (lamina II) of the spinal dorsal horn [13-17]. Pain information carried by small primary afferent fibers is processed and integrated by lamina II nociceptive neurons. Therefore, the substantia gelatinosa functions as the first relay station in the nociceptive pathway and plays a critical role in nociceptive transmission [16,17]. The excitability and activity of lamina II neurons is mainly regulated by glutamate released from primary nociceptive afferents [14,16,17]. Under pathological conditions, augmented glutamatergic transmission and the resulting enhanced excitability of substantia gelatinosa neurons are thought to cause hyperalgesia [18,19]. Immunohistochemical staining of CCL2 is most intense within the substantia gelatinosa, and CCL2 also co-localizes with substance P and CGRP-immunoreactive axon terminals in the outer portion of lamina II [2]. Following peripheral nerve injury, the central axon terminals of DRG neurons and activated astrocytes in the spinal dorsal horn release CCL2 [3,5]. Considering the physiological importance of lamina II neurons in processing the sensation of pain, it is very likely that during neuropathic pain, upregulated CCL2 in the superficial dorsal horn induces hyperalgesia by augmenting the excitatory glutamatergic transmission in substantia gelatinosa neurons and causing the central sensitization of lamina II neurons.

Several lines of evidence indicate that the spinal microglia plays an important role in the development of chronic pain [7,20-25]. Microglia in the spinal dorsal horn are converted from their resting shape to an activated shape after nerve injury or peripheral inflammation [20-22]. Inhibition of microglial activation attenuates the development of neuropathic pain [26]. Activated microglia produce and release various pro-inflammatory cytokines including tumor necrosis factor-α (TNF-α) [27-29]. Pro-inflammatory cytokines released by activated microglia could induce the central sensitization of substantia gelatinosa neurons by altering the excitatory or inhibitory synaptic transmission and cause hyperalgesia [30,31].

CCL2 is a key mediator for spinal microglia activation during neuropathic pain [7,12,23,24,29]. CCR2 is expressed in the spinal microglia [9]. When administered intrathecally, CCL2 causes microglial activation in the spinal dorsal horn of naive animals but not in CCR2-deficient mice [12,32]. Furthermore, a spinal injection of CCL2-neutralizing antibodies blocked microglial activation induced by nerve injury in the spinal dorsal horn [12,32]. Therefore, it is very likely that following peripheral nerve injury, an elevated CCL2 level within the spinal dorsal horn could cause the central sensitization of substantia gelatinosa neurons and enhance spinal nociceptive transmission by activating spinal microglia cells. In accord with this hypothesis, the present study provides evidence that minocycline, an inhibitor of microglial activation, blocks heat hyperalgesia induced by intrathecal injection of CCL2 and augmentation of glutamatergic transmission in substantia gelatinosa neurons. Our results also suggest that TNF-α mediates CCL2-induced thermal hyperalgesia and potentiation of glutamatergic transmission in lamina II neurons.

## Methods

### Experimental animals

Male Sprague–Dawley (SD) rats (BioLASCo Taiwan Co, Ltd), 2 months old, were used in the current study. The animals were housed under a 12 h light/dark cycle in a temperature-controlled room with *ad libitum* access to food and water. The animals were handled according to protocols approved by the Animal Care and Use Committee of Chang Gung University.

### Drugs

Recombinant rat CCL2 was obtained from R&D Systems (Minneapolis, MN, USA). Minocycline was purchased from Sigma (St Louis, MO, USA). BMS CCR2 22, picrotoxin, strychnine, tetrodotoxin, 2-amino-5-phosphonovalerate (APV) and 6-cyano-7-nitroquinoxaline-2,3-dione (CNQX) were obtained from Tocris Bioscience (Bristol, UK). WP9QY was purchased from Santa Cruz (Dallas, TX, USA).

### Intrathecal administration of CCL2

As in a previous study [11], 2-month-old male Sprague–Dawley rats were intrathecally administered with CCL2. Briefly, the rats were lightly anesthetized with isoflurane. Then, 1 μg of CCL2 (R & D Systems) dissolved in 10 μl PBS containing 0.1% BSA and protease inhibitor cocktail was intrathecally injected into the rats through an acute lumbar puncture between L4 and L5 using a 25-gauge stainless steel needle connected to a microsyringe. As a control, 10 μl of PBS containing 0.1% BSA and protease inhibitor cocktail was intrathecally administered to rats.

### Hot plate test for CCL2-induced heat hyperalgesia

As in a previous study [2], CCL2-induced thermal hyperalgesia was assessed using the hot plate test. Briefly, the animals were acclimatized to manipulations and behavioral apparatus (Model 7280 Hot Plate, Ugo Basile Biological Research Apparatus) for 2 days. The animals were then placed on a heated plate (50°C). The application of thermal stimuli to an animal caused the withdrawal of the hind paws, observed as licking of the hind paws. The latency value of hind-paw withdrawal due to a heat stimulus was recorded. For each experiment for measuring withdrawal latency, four latency measurements were taken at 10 min intervals to obtain a mean latency value.

### Whole-cell patch-clamp recordings in spinal cord slices

Whole-cell patch-clamp recordings of substantia gelatinosa neurons in a spinal cord slice were performed as for our previous studies [33,34]. Briefly, the rats were terminally anesthetized with sodium pentobarbital. The lumbar region of the spinal cord was removed and 350-μm-thick transverse slices were prepared using a vibratome slicer (VT 1000S, Leica) in an ice-cold extracellular solution containing: NaCl 80 mM, sucrose 75 mM, KCl 2.5 mM, MgCl_2_ 3.5 mM, NaH_2_PO_4_ 1.25 mM, CaCl_2_ 0.5 mM, NaHCO_3_ 26 mM, ascorbic acid 1.5 mM, sodium pyruvate 3 mM and kynurenic acid 0.01 mM. Before being used for patch-clamp recordings, the spinal cord slices were transferred to a holding chamber where they were submerged in artificial cerebrospinal fluid (ACSF) containing: NaCl 120 mM, KCl 2.4 mM, MgCl_2_ 2 mM, NaHCO_3_ 26 mM, CaCl_2_ 2 mM, NaH_2_PO_4_ 1.25 mM and glucose 15 mM. The ACSF was gassed with 95% O_2_/5% CO_2_ for 1 hour.

Following the recovery period, a spinal cord slice was transferred to a recording chamber mounted on the stage of an upright microscope (Axioskop, Zeiss) and superfused continuously with ACSF pre-gassed at room temperature with 95% O_2_/5% CO_2_. Neurons in the outer portion of lamina II of the spinal dorsal horn were visualized using a 40× water immersion objective (Zeiss) with the aid of differential interference contrast optics and an infrared-sensitive camera (Newvicon C2400-79, Hamamatsu). The neurons were used for whole-cell patch-clamp recordings. Patch pipets with a resistance of 5 MΩ were filled with a solution containing: KCl 60 mM, KF 65 mM, MgCl_2_ 2 mM, HEPES 10 mM, EGTA 1 mM, CaCl_2_ 0.1 mM_,_ ATP 2 mM and GTP 0.5 mM at pH = 7.3. The membrane currents or potentials recorded by the patch-clamp amplifier (Axopatch-200B, Axon Instruments) were filtered, digitized (Digidata 1200, Axon Instruments) and stored for later analysis. The series resistance was usually <15 MΩ. The holding potentials, data acquisition and analysis were controlled by the software pCLAMP 8.0 (Axon Instruments). Whole-cell patch-clamp recordings were performed at room temperature (25°C to 26°C).

In the presence of the GABA_A_ receptor antagonist picrotoxin (100 μM) and the glycine receptor antagonist strychnine (5 μM), a concentric bipolar stimulating electrode was placed at the dorsal root entry zone. Constant-current electrical stimuli (0.2 ms, 1.5 mA to 3 mA, and 0.2 Hz), adequate for high-threshold nociceptive afferent excitation in a rat spinal slice, were used to evoke monosynaptic glutamatergic excitatory postsynaptic currents (EPSCs) [35,36]. The peak amplitudes of ten consecutive evoked EPSCs in each experiment were averaged at 0.2 Hz. To record spontaneous miniature EPSCs (mEPSCs), picrotoxin (100 μM), strychnine (5 μM) and the Na^+^ channel blocker tetrodotoxin (TTX; 0.5 μM) were added to the external solution. Spontaneous GABAergic miniature inhibitory postsynaptic currents (mIPSCs) were recorded in the presence of the NMDA receptor antagonist 2-amino-5-phosphonovalerate (50 μM), the AMPA receptor antagonist 6-cyano-7-nitroquinoxaline-2,3-dione (CNQX; 25 μM), strychnine (5 μM) and TTX (0.5 μM). The amplitude and frequency of mEPSCs and mIPSCs were analyzed by the Mini-Analysis program (Synaptosoft).

### Statistics

All results are expressed as the mean ± standard error of *n* experiments. Statistical significance among multiple experimental groups was determined by a one-way ANOVA followed by a Dunnett’s test. An unpaired Student’s *t*-test (two-tailed) was used to determine whether there was a significant difference between two groups of data *P* < 0.01 was considered significant.

## Results

### Intrathecal administration of CCL2 induces heat hyperalgesia

**Figure 1:**
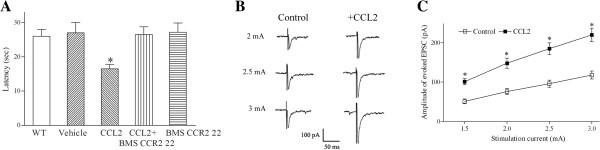
**Intrathecal administration of CCL2 induces heat hyperalgesia and increases the magnitude of evoked glutamatergic EPSCs of substantia gelatinosa neurons. (A)** CCL2 (1 μg) was intrathecally injected into 2-month-old rats. One day after the injection, the hot plate assay was used to measure hind-paw withdrawal latency. As a control, the vehicle was intrathecally injected into rats. Compared to wild-type (WT) rats and rats intrathecally administrated with the vehicle, the latency of hind-paw withdrawal caused by the heat stimulus was significantly reduced in the rats intrathecally injected with CCL2. Note that co-administration of CCR2 antagonist BMS CCR2 22 with CCL2 completely blocked CCL2-induced thermal hyperalgesia. Each bar shows the mean ± standard error for 12 or 20 rats. *P < 0.01 compared to wild-type rats. **(B)** One day after intrathecally injecting the vehicle or CCL2 (1 μg) into the rats, glutamatergic EPSCs were evoked by a stimulating electrode placed at the dorsal root entry zone and recorded from an outer lamina II neuron in a spinal cord slice prepared from a vehicle- or CCL2-treated rat. Compared to representative EPSCs evoked by various magnitudes of stimulation currents for control rats, the amplitude of representative EPSCs was significantly higher for lamina II neurons of CCL2-treated rats. **(C)** Slope of input–output curve for evoked EPSCs was significantly increased in substantia gelatinosa neurons of CCL2-injected rats. Each point represents the mean ± standard error for 13 to 15 neurons from seven rats. Holding potential (*V*_*H*_) was -60 mV. *P < 0.01 compared to control neurons.

Two-month-old rats were intrathecally administered with 1 μg of CCL2 dissolved in PBS containing protease inhibitor cocktail (*n* = 20). As a control, the vehicle was intrathecally injected into the animals. Then, the hot plate test was to verify CCL2-induced thermal hyperalgesia. Compared to wild-type or vehicle-injected rats (*n* = 20), the latency of hind-paw withdrawal caused by the heat stimulus was significantly reduced 1 day after the intrathecal injection of CCL2 (Figure 1A). This finding shows that intrathecal administration of CCL2 enhances nociceptive transmission in the spinal dorsal horn and causes heat hyperalgesia.

Intrathecal injection of a potent and specific CCR2 antagonist BMS CCR2 22 (3 μg) [37] did not affect the latency of hind-paw withdrawal in the hot plate test (Figure 1A; *n* = 12 animals). However, co-administration of CCR2 antagonist BMS CCR2 22 (3 μg) with CCL2 (1 μg) completely blocked the CCL2-induced reduction in the latency of hind-paw withdrawal and thermal hyperalgesia (Figure 1A; *n* = 12 animals), indicating that CCR2 receptors are involved in CCL2-mediated hyperalgesia in the spinal dorsal horn.

### Intrathecal injection of CCL2 strengthens excitatory glutamatergic transmission by substantia gelatinosa neurons via a presynaptic mechanism

The augmented excitatory glutamatergic transmission in substantia gelatinosa (lamina II) neurons could lead to enhanced nociceptive transmission in the spinal dorsal horn and resulting hyperalgesia [18,19]. A high level of CCL2 immunohistochemical staining has been found in the outer portion of lamina II of the spinal dorsal horn [2]. Therefore, it was hypothesized that intrathecal injection of CCL2 causes thermal hyperalgesia by strengthening glutamatergic transmission in substantia gelatinosa neurons and facilitating pain transmission in the spinal dorsal horn. To test this hypothesis, 1 day after intrathecally injecting the vehicle or CCL2 (1 μg) into rats, nociceptive afferent-mediated monosynaptic glutamatergic EPSCs, evoked by a stimulating electrode placed at the dorsal root entry zone, were recorded from outer lamina II neurons in spinal cord slices prepared from control vehicle- or CCL2-treated rats (Figure 1B). The mean resting membrane potential, membrane capacitance and input resistance of control outer lamina II neurons were -60.3 ± 2 mV, 28.9 ± 0.9 pF and 245.9 ± 23.2 MΩ, respectively (*n* = 15 neurons from 7 rats). Intrathecal injection of CCL2 (1 μg) did not significantly affect the resting membrane potential (-59.4 ± 1 mV; *n* = 15 neurons from 7 rats), membrane capacitance (31.8 ± 1.9 pF) or input resistance (271.8 ± 21.5 MΩ) of lamina II neurons.

Compared to the evoked glutamatergic EPSCs of outer lamina II neurons recorded vehicle-treated rats, 1 day after the intrathecal administration of CCL2 (1 μg), the amplitude of the evoked EPSCs and the slope of the input–output curve for the evoked EPSCs were significantly increased in lamina II neurons of CCL2-treated rats (Figure 1B,C). These results suggest that intrathecal injection of CCL2 augments glutamatergic transmission in substantia gelatinosa neurons and causes the central sensitization of lamina II neurons.

**Figure 2:**
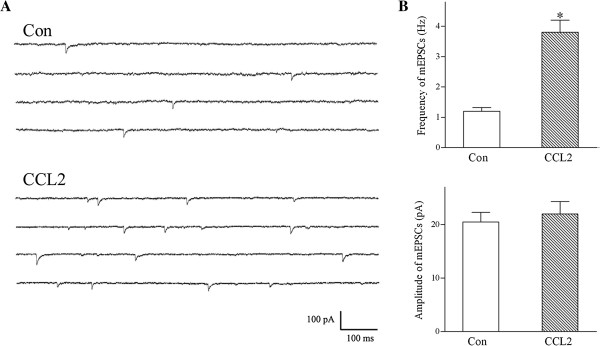
**Intrathecal injection of CCL2 strengthens glutamatergic transmission in lamina II neurons via a presynaptic mechanism. (A)** One day after intrathecally injecting vehicle or CCL2 (1 μg) into the rats, spontaneous mEPSCs were recorded from outer lamina II neurons in spinal cord slices. Compared to a control lamina II neuron, the frequency of spontaneous mEPSCs was significantly increased in a lamina II neuron from a CCL2-injected rat. *V*_*H*_ = -60 mV. **(B)** Intrathecal administration of CCL2 (1 μg) greatly increased the frequency of mEPSCs in substantia gelatinosa neurons without affecting the mean amplitude. Each bar shows mean ± standard error for 12 neurons from 6 rats. *P < 0.01 compared to control neurons. Con, control; mEPSC, miniature excitatory postsynaptic current.

To determine whether CCL2 enhances glutamatergic neurotransmission by a presynaptic or postsynaptic mechanism, we recorded spontaneous glutamatergic miniature EPSCs (mEPSCs) from lamina II neurons of control and CCL2-treated rats (Figure 2). One day after intrathecal administration of CCL2 (1 μg), the frequency of spontaneous glutamatergic mEPSCs was greatly increased in lamina II neurons from CCL2-treated animals (Figure 2A,B). Intrathecal injection of CCL2 did not affect the mean amplitude of mEPSCs (Figure 2A,B). These results suggest that intrathecal administration of CCL2 strengthens excitatory glutamatergic transmission lamina II neurons through a presynaptic mechanism.

We also recorded spontaneous GABAergic mIPSCs from outer lamina II neurons from vehicle- or CCL2-injected rats. Compared to lamina II neurons from vehicle-treated animals, intrathecal injection of CCL2 failed to affect the frequency (control frequency was 1.5 ± 0.2 Hz; with CCL2, frequency was 1.4 ± 0.1 Hz; *n* = 7 neurons from 4 rats) or amplitude (control magnitude was 22.5 ± 2.5 pA; with CCL2, amplitude was 21.8 ± 2.1 pA; *n* = 7 neurons from 4 animals) of spontaneous GABAergic mIPSCs in lamina II neurons.

### Minocycline inhibits spinal CCL2-induced thermal hyperalgesia and potentiation of glutamatergic transmission in substantia gelatinosa neurons

During neuropathic pain, microglial activation plays an important role in the central sensitization of nociceptive transmission in the spinal dorsal horn [23-25]. CCL2 is a key mediator of spinal microglia activation during neuropathic pain [7,12,23,24,29], and intrathecal injection of CCL2 induces microglial activation in the spinal dorsal horn of naive animals [12,32]. Therefore, we hypothesized that intrathecal administration of CCL2 would enhance spinal nociceptive transmission and cause thermal hyperalgesia by activating the spinal microglia. This hypothesis was tested using intrathecal administration of minocycline, a specific inhibitor of microglial activation [38,39].

**Figure 3:**
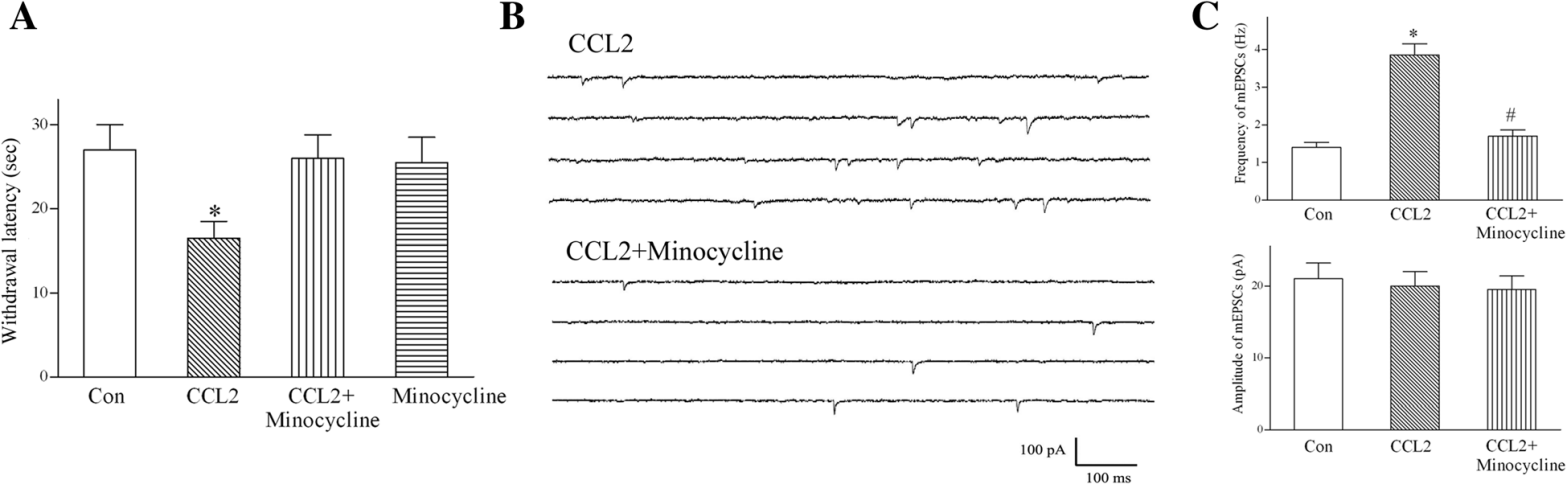
**Minocycline inhibits CCL2-induced heat hyperalgesia and blocks CCL2-induced enhancement of glutamatergic transmission in substantia gelatinosa neurons. (A)** Intrathecal administration of CCL2 (1 μg) decreased hind-paw withdrawal latency and caused heat hyperalgesia. Intrathecal co-injection of minocycline (100 μg) and CCL2 (1 μg) completely blocked the CCL2-induced reduction in the latency of hind-paw withdrawal and thermal hyperalgesia. Each bar shows mean ± standard error for 10 rats. *P < 0.01 compared to control rats. **(B)** One day after intrathecal administration of CCL2 (1 μg), the frequency of spontaneous mEPSCs was greatly increased a lamina II neuron. Intrathecal co-injection of CCL2 (1 μg) and minocycline (100 μg) inhibited the CCL2-induced increase in the frequency of mEPSCs in a lamina II neuron. *V*_*H*_ = -60 mV. **(C)** After intrathecal co-administration with minocycline, CCL2 did not significantly increase the frequency of mEPSCs in substantia gelatinosa neurons. Each bar shows mean ± standard error for 12 neurons from 6 rats. *P < 0.01 compared to control neurons. ^#^*P* < 0.01 compared to CCL2-treated neurons. Con, control; mEPSC, miniature excitatory postsynaptic current.

Previous studies [40,41] showed that an intrathecal injection of minocycline (100 μg) blocked microglial activation. Intrathecal administration of minocycline alone did not affect the latency of hind-paw withdrawal (Figure 3A). In accord with our hypothesis that CCL2 causes heat hyperalgesia by activating the microglia, hot plate assays showed that intrathecal co-injection of minocycline (100 μg) and CCL2 (1 μg) completely blocked the CCL2-induced reduction in the latency of hind-paw withdrawal and thermal hyperalgesia (Figure 3A).

An activated microglia releases pro-inflammatory cytokines, which could alter glutamatergic transmission by dorsal horn lamina II neurons [30,31]. Thus, it is possible that CCL2 enhances glutamatergic transmission in substantia gelatinosa neurons by activating the spinal microglia. Consistent with this hypothesis, following intrathecal co-administration of CCL2 (1 μg) and minocycline (100 μg), CCL2 failed to increase the frequency of glutamatergic mEPSCs and failed to promote glutamine release by outer lamina II neurons (Figure 3B,C).

### Intrathecal injection of CCL2 enhances glutamatergic transmission of substantia gelatinosa neurons and causes thermal hyperalgesia via TNF-α

In the present study, our results suggest that intrathecal injection of CCL2 potentiates glutamatergic transmission in substantia gelatinosa neurons and causes thermal hyperalgesia by activating the spinal microglia. An activated microglia releases the pro-inflammatory cytokine tumor necrosis factor-α (TNF-α) [27-29]. As with the CCL2 effect observed in this study, TNF-α has been shown to increase the frequency of glutamatergic mEPSCs in lamina II neurons and enhance excitatory synaptic transmission in the spinal dorsal horn [30,31]. CCL2 activation of the spinal microglia is believed to cause the synthesis and release of TNF-α [7,29]. Therefore, we hypothesized that intrathecal administration of CCL2 would augment glutamatergic transmission by substantia gelatinosa neurons and cause heat hyperalgesia via TNF-α. This hypothesis was tested using intrathecal injection of WP9QY, a selective TNF-α antagonist [42].

**Figure 4:**
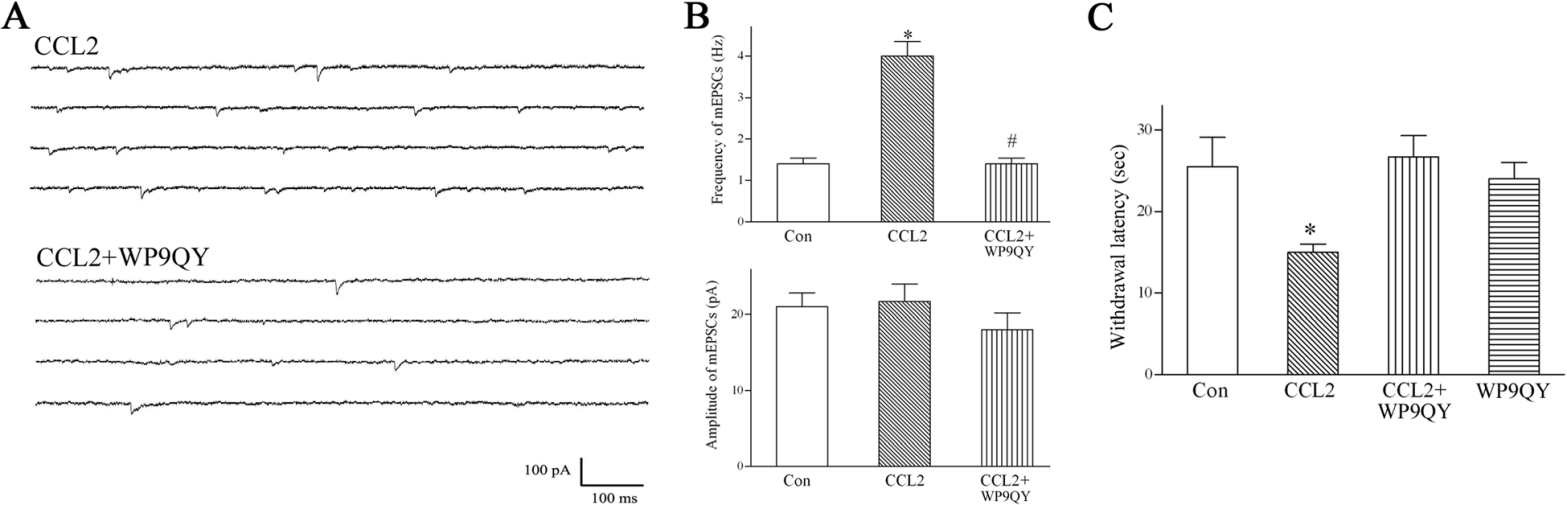
**TNF-α antagonist WP9QY blocks CCL2-induced enhancement of excitatory synaptic transmission in substantia gelatinosa neurons and inhibits CCL2-induced heat hyperalgesia. (A)** Intrathecal administration of CCL2 increased the frequency of spontaneous mEPSCs in a lamina II neuron. Intrathecal co-injection of WP9QY and CCL2 completely inhibited the CCL2-induced increase in the frequency of mEPSCs in a lamina II neuron. *V*_*H*_ = -60 mV. **(B)** After co-administration with WP9QY, CCL2 failed to increase the frequency of mEPSCs in substantia gelatinosa neurons. Each bar shows mean ± standard error for 12 neurons from 6 rats. *P < 0.01 compared to control neurons. ^#^*P* < 0.01 compared to CCL2-treated neurons. **(C)** Intrathecal injection of CCL2 (1 μg) decreased hind-paw withdrawal latency and caused thermal hyperalgesia. Intrathecal co-administration of WP9QY and CCL2 completely inhibited CCL-induced heat hyperalgesia. Each bar represents mean ± standard error for ten rats. *P < 0.01 compared to control rats. Con, control; mEPSC, miniature excitatory postsynaptic current.

Following intrathecal co-administration of CCL2 (1 μg) and WP9QY (20 μg), CCL2 failed to increase the frequency of glutamatergic mEPSCs and potentiate glutamatergic transmission by substantia gelatinosa neurons (Figure 4A,B). Hot plate assays also showed that intrathecal co-injection of WP9QY (20 μg) and CCL2 (1 μg) completely blocked CCL2-induced reduction in the latency of hind-paw withdrawal and thermal hyperalgesia (Figure 4C).

## Discussion

Multiple lines of evidence suggest that following a peripheral nerve injury, chemokine CCL2 is involved in mediating the hyperalgesia of neuropathic pain in the dorsal root ganglion (DRG) and spinal dorsal horn [1-7,43-48]. A peripheral nerve injury upregulates the expression of CCL2 and its receptor CCR2 in the DRG and spinal dorsal horn [1,3,4,9,10,45-47]. Then, CCL2 induces hyperalgesia by causing peripheral sensitization of nociceptive DRG neurons and central sensitization of spinal dorsal horn neurons [6,7,48]. Our previous work suggested that CCL2 could facilitate pain transmission mediated by nociceptive DRG neurons and cause hyperalgesia by upregulating the expression and function of TRPV1 and Na_v_1.8 channels in DRG nociceptive neurons [49]. The exact mechanism by which CCL2 enhances nociceptive transmission in the spinal dorsal horn and induces the resulting hyperalgesia remains unknown.

In the present study, behavioral assays indicated that consistent with a previous study [2], intrathecal administration of CCL2 induced thermal hyperalgesia. Therefore, a rat intrathecally injected with CCL2 is a suitable animal model for investigating the cellular and molecular mechanisms by which CCL2 facilitates nociceptive processing by spinal dorsal horn neurons and the resulting heat hyperalgesia.

The substantia gelatinosa (lamina II) of the spinal dorsal horn receives nociceptive information from the peripheral organs through fine myelinated Aδ and unmyelinated C primary-afferent fibers [13-17]. The vast majority of substantia gelatinosa neurons are glutamatergic excitatory interneurons [50]. Nociceptive information is integrated by substantia gelatinosa neurons, which subsequently send a noxious sensation to the somatosensory cortex by exciting nociceptive projection neurons in lamina I and lamina V [13-17]. Therefore, the substantia gelatinosa plays an essential role in processing nociceptive information and is one of the key sites for generating central sensitization during neuropathic pain [18,19]. The excitability and activity of lamina II neurons arre mainly regulated by glutamate released from primary nociceptive afferents [14,16,17]. Under pathological conditions, strengthened glutamatergic transmission and the resulting augmented excitability of lamina II neurons could cause the central sensitization of substantia gelatinosa neurons, leading to enhanced nociceptive transmission in the spinal dorsal horn and hyperalgesia [18,19]. After peripheral nerve injury, the central nerve terminals of DRG neurons and activated astrocytes in the spinal dorsal horn release CCL2 [3,5]. Immunohistochemical staining of CCL2 is most intense in the outer portion of lamina II of the spinal dorsal horn [2]. Therefore, we hypothesized that during neuropathic pain, upregulated CCL2 in the spinal dorsal horn causes hyperalgesia by enhancing excitatory glutamatergic transmission in lamina II neurons and facilitating pain transmission in the spinal dorsal horn. In accord with our hypothesis, intrathecal administration of CCL2, which causes thermal hyperalgesia, increased the magnitude of evoked nociceptive afferent-mediated glutamatergic EPSCs recorded from outer lamina II neurons.

To determine whether CCL2 augments glutamatergic neurotransmission by either promoting glutamate release or potentiating the AMPA receptor-mediated postsynaptic response, spontaneous glutamatergic mEPSCs were recorded lamina II neurons of control and CCL2-treated rats. Intrathecal injection of CCL2 increased the frequency of spontaneous mEPSCs in lamina II neurons without affecting the mean amplitude. These results show that CCL2 facilitates pain transmission in the spinal dorsal horn and causes heat hyperalgesia by strengthening excitatory glutamatergic transmission by substantia gelatinosa neurons via a presynaptic mechanism. Interestingly, a recent study reported that peripheral nerve injury upregulated the expression of chemokine CCL1 in the spinal dorsal horn and that intrathecal administration of CCL1 induced hyperalgesia by enhancing glutamatergic neurotransmission in lamina II neurons via a presynaptic mechanism [51]. Therefore, facilitation of glutamate release and the resulting augmentation of glutamatergic synaptic transmission is likely to be one of the common pathogenic mechanisms by which chemokines induce the central sensitization of substantia gelatinosa neurons and cause neuropathic hyperalgesia.

Several lines of evidence indicate that microglial activation in the spinal dorsal horn contributes to the development and maintenance of neuropathic pain [7,20-25]. Peripheral nerve injury induces the activation of the microglia in the spinal dorsal horn [20-22]. An intrathecal injection of activated spinal cord microglia caused thermal hyperalgesia in naive animals [52]. Inhibition of microglial activation inhibits the development of neuropathic pain [26]. Activation of p38 MAPK induced through nerve injury in activated spinal microglia produces via transcriptional regulation various pro-inflammatory cytokines including TNF-α [27-29]. Pro-inflammatory cytokines released by activated microglia could cause the central sensitization of nociceptive dorsal horn neurons by altering excitatory or inhibitory synaptic transmission, leading to hyperalgesia [30,31].

Intrathecal administration of CCL2 induces microglial activation in the spinal dorsal horn [12,32]. CCL2 activates p38 MAPK in the microglia of the spinal dorsal horn [7,29,53]. Intrathecal injection of CCL2-neutralizing antibodies suppressed nerve-injury-induced activation of the spinal microglia [12,32]. Therefore, CCL2 is an important mediator of microglia activation in the spinal dorsal horn during neuropathic pain [7,12,23,24,29]. In the present study, we hypothesized that intrathecal injection of CCL2 would strengthen glutamatergic transmission by substantia gelatinosa neurons and cause heat hyperalgesia by inducing microglial activation. Consistent with our hypothesis, intrathecal co-administration of minocycline, a specific inhibitor of microglial activation, and CCL2 completely blocked CCL2-induced thermal hyperalgesia and blocked the augmentation of glutamatergic transmission substantia gelatinosa neurons. This finding strongly suggests that the elevation of CCL2 levels induced by peripheral nerve injury in the spinal dorsal horn causes the central sensitization of substantia gelatinosa neurons and facilitates spinal nociceptive transmission by activating the spinal microglia.

An activated microglia in the spinal dorsal horn enhances nociceptive transmission by releasing pro-inflammatory cytokines including TNF-α [27-29]. As with CCL2, intrathecal injection of TNF-α induces heat hyperalgesia [31]. TNF-α has also been shown to enhance excitatory synaptic transmission of the spinal dorsal horn by increasing the frequency of spontaneous glutamatergic EPSCs in substantia gelatinosa neurons [30,31]. Our results indicate that intrathecal administration of CCL2 causes thermal hyperalgesia and increases the frequency of spontaneous glutamatergic EPSCs without affecting inhibitory GABAergic transmission substantia gelatinosa neurons. In the present study, intrathecal co-administration of WP9QY, a selective TNF-α antagonist, and CCL2 completely blocked CCL2 augmentation of glutamatergic transmission in substantia gelatinosa neurons and CCL2-induced thermal hyperalgesia. Therefore, it is possible that intrathecal injection of CCL2 activates spinal microglia and causes the release of TNF-α, which subsequently augments excitatory glutamatergic transmission in substantia gelatinosa neurons and mediates CCL2-induced heat hyperalgesia.

## Conclusions

In summary, our results suggest that intrathecal injection of CCL2 causes thermal hyperalgesia by augmenting excitatory glutamatergic transmission in substantia gelatinosa neurons through a presynaptic mechanism and facilitating nociceptive transmission in the spinal dorsal horn. Intrathecal co-administration of minocycline, a specific inhibitor of microglial activation, or WP9QY, a selective TNF-α antagonist, completely inhibited CCL2 potentiation of glutamatergic transmission substantia in gelatinosa neurons and CCL2-induced heat hyperalgesia. These results suggest that upregulation of spinal CCL2 induced by peripheral nerve injury causes the central sensitization of substantia gelatinosa neurons by activating spinal microglia and that TNF-α mediates CCL2-induced thermal hyperalgesia and augmentation of glutamatergic transmission in lamina II neurons.

## Abbreviations

ACSF: Artificial cerebrospinal fluid; BSA: Bovine serum albumin; CCL2: CC chemokine ligand 2; CGRP: Calcitonin gene-related peptide; DHSC: Dorsal horn of the spinal cord; DRG: Dorsal root ganglion; EPSC: Excitatory postsynaptic current; mEPSC: Miniature EPSC; mIPSC: Miniature inhibitory postsynaptic current; PBS: Phosphate-buffered saline; SP: Substance P; TNF-α: Tumor necrosis factor-α; TRPV1: Transient receptor potential vanilloid receptor 1; TTX: Tetrodotoxin; WT: Wild type.

## Competing interests

The authors declare that they have no competing interests.

## Authors’ contributions

HLW, AHL and YLC designed the study. CYH, YLC and JCL performed the experiments. HLW, JCL, AHL, YLC and CYH discussed the results and prepared the manuscript. All authors have read and approved the final version of this manuscript.
